# Bacterial isolates of early-onset neonatal sepsis and their antibiotic susceptibility pattern between 1998 and 2004: an audit from a center in India

**DOI:** 10.1186/1824-7288-37-32

**Published:** 2011-07-11

**Authors:** Ramesh Bhat Y, Leslie Edward S Lewis, Vandana KE

**Affiliations:** 1 Department of Pediatrics, Kasturba Medical College, Manipal University, Manipal-576104. Udupi District, Karnataka, India; 2 Department of Microbiology, Kasturba Medical College, Manipal University, Manipal-576104. Udupi District, Karnataka, India

**Keywords:** Early onset sepsis, neonates, blood culture isolates, antibiotic susceptibility

## Abstract

**Background:**

Epidemiology and surveillance of neonatal sepsis helps in implementation of rational empirical antibiotic strategy.

**Objective:**

To study the frequency of bacterial isolates of early onset neonatal sepsis (EONS) and their sensitivity pattern.

**Methods:**

In this retrospective study, a case of EONS was defined as an infant who had clinical signs or born to mothers with potential risk factors for infection, in whom blood culture obtained within 72 hours of life, grew a bacterial pathogen. Blood culture sample included a single sample from peripheral vein or artery. Relevant data was obtained from the unit register or neonatal case records.

**Results:**

Of 2182 neonates screened, there were 389 (17.8%) positive blood cultures. After excluding coagulase-negative Staphylococci (160), we identified 229 EONS cases. Preterm neonates were 40.6% and small for gestational age, 18.3%. Mean birth weight and male to female ratio were 2344.5 (696.9) g and 1.16:1 respectively. Gram negative species represented 90.8% of culture isolates. Pseudomonas (33.2%) and Klebsiella (31.4%) were common among them. Other pathogens included Acinetobacter (14.4%), Staphylococcus aureus (9.2%), E.coli (4.4%), Enterobacter (2.2%), Citrobacter (3.1%) and Enterococci (2.2%). In Gram negative group, best susceptibility was to Amikacin (74.5%), followed by other aminoglycosides, ciprofloxacin and cefotaxime. The susceptibility was remarkably low to ampicillin (8.4%). Gram positive group had susceptibility of 42.9% to erythromycin, 47.6% to ciprofloxacin and above 50% to aminoglycosides. Of all isolates, 83.8% were susceptible to either cefotaxime or amikacin

**Conclusion:**

Gram-negative species especially Pseudomonas and Klebsiella were the predominant causative organisms. Initial empirical choice of cefotaxime in combination with amikacin appeared to be rational choice for a given cohort.

## Background

Early onset bacterial infection places the neonate at risk of death and long term morbidity [1-,^3^]. Improvement in outcome and successful treatment depends on early initiation of appropriate antibiotic therapy. The pattern of causative organisms has been constantly changing [4] and the frequent emergence of resistant bacteria [5] compounds the problem further. This highlights the need for surveillance of sepsis for optimum therapy. Knowledge of likely causative organisms and their antimicrobial sensitivity pattern could aid in choosing prompt and appropriate therapy for early-onset neonatal sepsis (EONS). The epidemiology of EONS in the developed and developing countries shows some important differences in the pattern of etiological bacteria and their antibiotic susceptibility [1-3,6,7]. In developed countries, Group B Streptococcus (GBS) was the common etiological agent for EONS [6,7]. Following adoption of preventive strategies for GBS, Escherichia coli (E.coli) was identified as predominant pathogen [7,8]. Developing nations reported an entirely a different bacterial spectrum [3,7,9-11]. Current study was undertaken to find out the common bacterial pathogens and their susceptibility pattern in neonates with early onset sepsis in a tertiary care hospital providing neonatal intensive care services.

## Methods

Present study was carried out in a neonatal unit of Kasturba Hospital, Kasturba Medical College, Manipal from January 1998 to December 2004. We retrospectively evaluated the case records of neonates who had EONS. A case of EONS was defined as an infant who had clinical signs of sepsis or those who were born to mothers with potential risk factors for infection, in whom blood culture obtained within 72 hours of life, grew a bacterial pathogen. Risk factors in the mother included prolonged rupture of membranes (PROM) of >12hours, fever, urinary tract infection, chorioamnionitis and meconium stained amniotic fluid. Blood culture sample included a single sample collected from a peripheral vein or artery under aseptic conditions. The local site was cleansed with 70% alcohol and povidone iodine (1%) followed by 70% alcohol again. Blood cultures were done in Brain Heart Infusion biphasic medium. Subcultures were done on Sheep blood agar and MacConkey agar at the earliest visual detection of turbidity or blindly on days 1, 4 and 7 if the bottles did not show turbidity. Gram's staining was performed on the bottles showing turbidity. Bacterial isolates were identified and antimicrobial susceptibility test was performed using Kirbey Bauer disc diffusion method. (Reference -National Committee for Clinical Laboratory Standards. Performance standards for antimicrobial disk susceptibility tests; Approved standard. 7th ed. NCCLS Document M2 - A7. Wayne, PA: National Committee for Clinical Laboratory Standards, 2000.) After obtaining blood for culture, the neonates were administered intravenous ampicillin and gentamicin as the first line of antibiotics. Antibiotic therapy was continued or changed, based on the isolation of organisms in the blood culture and their sensitivity pattern. Other neonatal intensive care and support were given as required. CSF analysis was done when there was clinical suspicion of meningitis and in whom blood culture grew microorganism. Urine and other cultures were obtained on indications. The demographic data, blood culture reports, organisms and their antibiotic susceptibility were obtained from the unit register and/or neonatal case records. Neonates with blood cultures that grew only coagulase-negative staphylococcus were excluded. Data was analyzed using Statistical Package for Social Sciences (SPSS) version 11.5 software.

## Results

**Figure 1:**
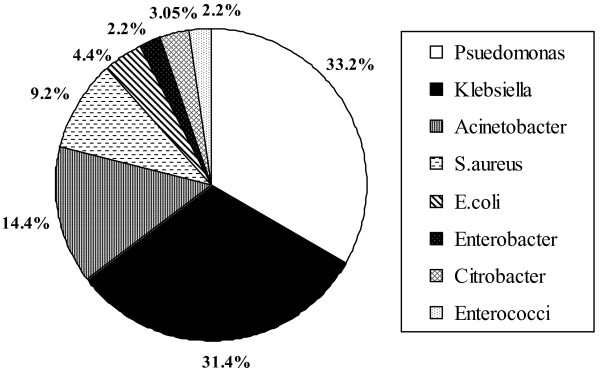
Bacterial isolates of early onset sepsis (n = 229)

Of 2182 neonates screened for EONS, there were 389 (17.8%) positive blood cultures. After excluding growth of coagulase-negative Staphylococci (160 cases, 41.1%), we identified 229 cases of EONS. Both blood and CSF cultures were positive in 8(3.5%) cases. Demographic characteristics of 229 neonates were shown in Table 1. The male to female ratio was 1.16:1. The predisposing factors and comorbid conditions were shown in table 2. Obstetric risk factors and preterm delivery was present in 54.6% (125/229). Figure 1 shows the distribution of bacterial isolates from blood cultures. During the 7 years period, 208 (90.8%) sepsis cases were attributable to Gram negative species and 21 (9.2%) were attributable to Gram positive species. Pseudomonas spp (33.2% of all isolates, 36.5% of Gram-negatives) and Klebsiella pneumoniae (31.4% of all isolates, 34.6% of Gram-negative isolates) were the common isolates that accounted for nearly 2/3^rd ^of total blood culture isolates. Acinetobacter spp (14.4%) was the third common organism. Staphylococcus aureus represented 9.2%( 21/229). Other pathogens included E.coli (10), Enterobacter spp (5), Citrobacter spp (7) and Enterococcus spp (5).

**Table 1:** Demographic characteristics (n = 229)

Birth weight, mean (SD)	2344.5(696.9) g
Male	123 (53.7%)

Female	106 (46.3%)

Term	136 (59.4%)

Preterm	93 (40.6%)

Small for gestational age	42 (18.3%)

**Table 2:** Predisposing factors and comorbidity among blood culture positive neonates (n = 229)

	Number	%
1. Obstetric risk factors		

PROM	11	4.8

Chorioamnionitis	8	3.5

MSAF	13	5.7

2. Neonatal factors		

Prematurity alone	47	20.5

Perinatal asphyxia	10	4.4

RDS	14	6.1

Respiratory distress	19	8.3

MAS	38	16.6

Meningitis	8	3.5

Hypoglycemia	6	2.6

Congenital malformations	13	5.7

PROM, prolonged rupture of membranes; MSAF, meconium stained amniotic fluid; RDS, respiratory distress syndrome; MAS, meconium aspiration syndrome.

Table 3 shows the antibiotic susceptibility pattern of different bacterial isolates. Pseudomonas species were mostly susceptible to amikacin, moderately to gentamicin, netilmicin and ciprofloxacin, and less susceptible to ceftazidime and piperacillin. Klebsiella and Acinetobacter species were more susceptible to amikacin and moderately to other aminoglycosides, third generation cephalosporins and ciprofloxacin. E.coli and other gram negative bacilli were susceptible to amikacin and netilmicin but remarkably less sensitive to ampicillin. S.aureus was better susceptible to erythromycin, ciprofloxacin, vancomycin and amikacin when compared to ampicillin and cefotaxime. All Enterococci were susceptible to vancomycin. There were three methicillin resistant Staphylococci and they were susceptible to vancomycin.

**Table 3:** Antibiotic susceptibility pattern of blood culture isolates (% susceptible)

	Pseudomonas	Klebsiella	Acineto-bacter	S.aureus	E.coli	Entero-bacter	Citro-bacter	Entero-cocci
Ampicillin	NT	6.9	33.3	28.6	20	0	12.5	20

Gentamicin	56.6	25	48.5	61.9	40	0	25	20

Amikacin	76.3	77.8	66.7	76.2	70	100	50	NT

Netilmycin	56.6	31.9	66.7	52.4	30	40	75	NT

Tobramycin	26.3	6.9	33.3	NT	20	40	25	NT

TMP/SMX	NT	33.3	21.2	28.5	40	20	25	0

Ciprofloxacin	40.8	51.4	36.4	47.6	20	80	50	40

Piperacillin	23.7	NT	NT	NT	NT	NT	NT	NT

Vancomycin	NT	NT	NT	100	NT	NT	NT	100

Cefuroxime	NT	5.6	6.1	14.2	7	8	12.5	NT

Cefotaxime	NT	43	27.3	28.5	40	20	37.5	NT

Ceftazidime	14.5	45.8	36.3	NT	10	0	12.5	NT

Erythromycin	NT	NT	NT	42.9	NT	NT	NT	NT

'NT' means not tested.

In Gram negative group, best overall sensitivity was to amikacin (74.5%). Gram positive group had sensitivity of 42.9% to erythromycin, 47.6% to ciprofloxacin and above 50% to aminoglycosides (Table 3 and Table 4)

**Table 4:** Susceptibility (%) of gram positive and gram negative organism groups

	Gram negative	Gram positive
Ampicillin	8.4	28.6

Gentamicin	40.4	65.3

Amikacin	74.5	61.7

Netilmycin	48.1	52.4

TMP/SMX	29.3	28.2

Ciprofloxacin	44.2	47.6

Vancomycin	100	100

Cefotaxime	32.7	28.5

Ceftazidime	27.9	NT

Piperacillin	23.7	NT

NT = not tested

## Discussion

We conducted an audit of positive blood cultures obtained from neonates with EONS over 7 years. The blood culture yield was about 18%. This is low compared to about 20% yield reported by Baltimore et al [8] and Gladstone et al [12]. Earlier, culture positive rate of 26% by Ahmed et al [11] and much higher rates of 51% by Karthikeyan [13] and 64% by Tallur et al [14] were reported. We used conventional blood culture techniques in the present study. The automated systems like BacT alert may provide the results earlier and improve the yield [15]. Coagulase-negative Staphylococcus (CONS) has been identified as causative organism for EONS. In a study by Agarwal et al [16], approximately two third of CONS sepsis has developed within first three days of life. We excluded CONS from analysis because we used single blood sample for culture and it was difficult to analyse the response to antibiotics from the retrospective nature of the study, although discriminating true CONS infection from contamination, from single culture has been recognized [17]. Mothers received amoxicillin injections whenever indicated to reduce neonatal sepsis.

A male predominance was found in our study which agrees with previous reports [2,11,18-20]. Among the neonates in whom blood culture was positive, prematurity and obstetric factors were present in 54.6% (125/229). Schuchat et al [21] found an obstetric risk factor-preterm delivery, intrapartum fever, or membrane rupture >/ = 18 hours in 49% of GBS and 79% of other sepsis. In an attempt to identify possible risk factors for EONS, Bizzarro et al [19] found preterm labor in 43%, fever before delivery in 26%, prolonged rupture of membranes in 46% and chorioamnionitis in 20%. A significant association of EONS with prolonged rupture of membranes and foul smelling liquor was found by Chacko and Sohi [3]. They also found among infants at risk of EOS, 20.6% developed sepsis compared to only 0.5% of those without these risk factors. Tallur et al [14] reported association of PROM > 24 hours in 14% and perinatal asphyxia in 22%. Association of meconium stained amniotic fluid with sepsis was identified by Kuruvilla et al [9]. Agarwal et al [16] found EONS more frequently in neonates with perinatal asphyxia.

Bacteria causing neonatal sepsis continue to change [4-10,19,22,23]. They also differ from developed to developing country and place to place. In industrialized world GBS caused EONS predominantly [2,6,7,21,22,24,25]. E.coli was 2^nd ^most common. Following GBS prophylaxis, decreasing incidence of GBS [5,7,8,26] and increased rate of E.coli infections [5,7,8] have been reported. Significant proportions (50%-37%) of EONS due to gram positive organisms were reported from recent studies [6-8]. In contrast, 90.2% of isolates were gram negative species in the present study. Predominance of gram negative isolates (67.2%-92.5%) has been reported by developing countries [7,8,11,18,27].

Following GBS prophylaxis, industrialized countries identified E.coli as the predominant organism for EONS. Developing countries also identified E.coli as the most common causative organism [9,10]. Rate of E.coli infection varied from 15.7%- 77.1%. Other studies from developing world found Klebsiella as the common organism [4,14]. Rate of Klebsiella infection varies from 8.9%-64%. In contrast, we identified Pseudomonas species as the predominant isolates. Similar observations were reported by Joshi et al [18] and Tallur et al [14,3]. Pseudomonas isolation rate varies from 8.9%-38.3%.

Acinetobacter accounted for 14.4% of EONS in our study. Infection rate up to 7.8% has been recognized [9,11,18]. Infection rates of other gram-negative organisms in the present study were similar to earlier reports [3,9,14]. The rate of S. aureus infection in the present study was 9.2%. Similar reports with rate of infection varying from 3.7%- 7% have been found previously [5,10,14]. However, Karthikeyan and Premkumar [13], in their analysis identified S aureus as a predominant pathogen (50% of EONS). A low rate of entrococci infection of present study is similar to the observations of Dobson and Baker [28].

### Antibiotic susceptibility

In the present study, Pseudomonas species were best susceptible to amikacin, moderately to gentamicin, netilmicin and ciprofloxacin, and less susceptible to ceftazidime and piperacillin. Above 60% sensitivity of the organism to aminoglycoside is recognized [14]. Lower susceptibility to amikacin (45%) was found by Agarwal et al [16]. In contrast, Pseudomonas aeruginosa in a study with controlled antibiotic programs by Cordero et al [6] remained fully susceptible to ceftazidime and gentamicin. Klebsiella and Acinetobacter species of present study were more susceptible to amikacin and moderately to other aminoglycosides, third generation cephalosporins and ciprofloxacin. Higher sensitivity to aminoglycosides was reported by Agarwal et al [16] and Kuruvilla et al [9]. Low sensitivity to ampicillin is similar in all these studies.

E.coli and other gram negative bacilli were susceptible to amikacin and netilmicin but remarkably less sensitive to ampicillin. High sensitivity (up to 93.7%) of E.coli to amikacin [14,16] and uniform susceptibility to cephalosporins have been described [14,19]. Low sensitivity of E.coli to ampicillin in the present study is similar to many earlier studies [5,14,19,21].

S.aureus of present study was better susceptible to erythromycin, ciprofloxacin, vancomycin and amikacin when compared to ampicillin and cefotaxime. Staphylococcal resistance of 79.3% to ampicillin [10] and low sensitivity to all commonly used antibiotics [13] were described in the literature. Susceptibility of Enterococci in the present study to aminoglycoside is similar to the observations of Dobson and Baker [28].

### Sensitivity of bacterial groups (Gram negative and Gram positive)

In the current study all isolates were best sensitive to amikacin while relatively less sensitive to other aminoglycosides. Good sensitivity of organisms to amikacin has been found by other researchers [10,14,16]. Some studies found good sensitivity to gentamicin [11,29,30]. The sensitivity was 17.5% to ampicillin. Cefotaxime had better sensitivity but only 32.3%. Low sensitivity of bacteria causing EONS to commonly used antibiotics has been found by other authors [10,14,19]. Tallur et al [14] reported that most isolates were resistant to ampicillin, gentamicin and cotrimaxazole. Ampicillin resistance of all Klebsiella isolates was reported by Thaver et al [31]. In contrast, good response to ampicillin has been found by Cordero et al [6] and to ampicillin-sulbactum by Mokuolu [27]. No increase in ampicillin resistant organisms following intrapartum antibiotics prophylaxis has also been reported [32]. Gram negative group had best overall sensitivity to Amikacin (74.5%), followed by other aminoglycosides and ciprofloxacin. Cephalosporins had low sensitivity. Higher sensitivity of gram negative group to gentamicin and ceftazidime has been reported by Mokuolu [27] and good sensitivity to cephalosporins by others [11,14]. Meropenem sensitivity was tested since 2003. Gram negative organisms were universally susceptible to meropenem.

Of all isolates, 83.8% (192/229) were sensitive to either cefotaxime or amikacin and hence using this combination as the initial choice while awaiting blood culture reports seems reasonable. However, these results are limited to study cohorts and every center should have its own control on their bacterial strains.

## Conclusion

Bacterial spectrum for EONS could be different in developed and developing countries. Continued surveillance of neonatal sepsis is mandatory for each center due to temporal changes in the causative organisms and their antibiotic susceptibility. Periodic evaluations not only show the trend of increasing resistance to commonly used antibiotics but also help in implementation of a rational empirical treatment strategy. Present study indicated that Gram-negative species continue to be the predominant causative organisms among study cohorts. Pseudomonas and Klebsiella played a major role and, Acinetobacter, Staphylococci and E.coli contributed to the rest. A low susceptibility to commonly used antibiotics like ampicillin is a cause for concern. The antibiotic susceptibility profiles suggested that for a given cohort, initial empirical choice of cefotaxime in combination with amikacin was the most rational.

## Conflicts of interest

Authors declare that they have no conflicts of interest

## Authors' contributions

RB involved in study design, data collection, analysis, treatment of cases, manuscript preparation and draft. VKE involved in blood culture analysis, expert microbiological inputs and approval of the manuscript. LEL is involved in treatment of cases and manuscript preparation. All authors have read and approved the final manuscript.
